# Toothbrush Design and Developmental Characteristics Influencing Plaque Removal in 7‐Year‐Old Children: A Randomized Crossover Trial

**DOI:** 10.1111/ipd.70137

**Published:** 2026-08-10

**Authors:** Ece Baysan Maşızade, Ezgi Baltacı, Nazlı Durmuş, Selçuk Korkmaz, Neslihan Özveren

**Affiliations:** ^1^ Department of Paediatric Dentistry, Faculty of Dentistry Trakya University Edirne Türkiye; ^2^ Department of Paediatric Dentistry, Faculty of Dentistry Mersin University Mersin Türkiye; ^3^ Department of Biostatistics and Medical Informatics, Faculty of Medicine Trakya University Edirne Türkiye

**Keywords:** child development, dental plaque, motor skills, pediatric dentistry, powered toothbrush, triple‐headed toothbrush

## Abstract

**Background:**

Evidence comparing toothbrush designs and the influence of children's developmental abilities on brushing performance is limited.

**Aim:**

To compare the plaque‐removal efficacy of three toothbrush types in 7‐year‐old children and to assess whether developmental characteristics affect brushing performance.

**Design:**

This single‐blind, randomized, three‐period crossover clinical trial included 60 typically developing 7‐year‐old children. Participants used a conventional manual, triple‐headed manual, and rechargeable powered toothbrush in randomized order with washout periods. Plaque was assessed using the modified Quigley–Hein Plaque Index and the Approximal Plaque Index. Developmental characteristics were evaluated using the parent‐reported Five‐to‐Fifteen questionnaire.

**Results:**

Mean plaque reduction differed significantly among toothbrush types. Powered (1.833 ± 0.705) and triple‐headed manual toothbrushes (1.802 ± 0.678) achieved greater plaque reduction than the conventional manual toothbrush (1.310 ± 0.563; both *p* < 0.001), with no difference between powered and triple‐headed brushes (*p* = 0.963). Approximal plaque removal was highest with the powered toothbrush, reaching 75% complete removal on posterior vestibular surfaces (*p* ≤ 0.016). Children with gross motor difficulties showed lower reduction with the powered toothbrush than children without such difficulties (*p* = 0.048).

**Conclusion:**

Powered and triple‐headed toothbrushes are more effective than conventional manual brushes in 7‐year‐old children, with equivalent efficacy. Exploratory analyses suggest gross motor skills may influence powered toothbrush performance, warranting confirmation in adequately powered studies.

**Trial Registration:**

The study was registered retrospectively with the Australian New Zealand Clinical Trials Registry (ANZCTR; ACTRN12626000023358)

## Why This Paper Is Important to Pediatric Dentists

- Powered and triple‐headed toothbrushes provide significantly better plaque reduction than conventional manual toothbrushes in 7‐year‐old children, supporting their use as first‐line options in routine preventive care.
- Toothbrush efficacy is influenced by children's developmental abilities; notably, children with gross motor difficulties may achieve poorer plaque reduction with powered toothbrushes, highlighting the need for individualized recommendations.
- Incorporating simple developmental screening and parental guidance into oral‐hygiene instruction may help pediatric dentists tailor toothbrush selection and optimize plaque control outcomes in young children.

## 1 Introduction

Dental plaque, a biofilm of organized bacterial communities embedded in extracellular polymeric matrices, is a primary etiological factor in both dental caries and gingivitis in children [1]. Caries remains one of the most prevalent diseases among pediatric populations worldwide, with rates exceeding 50% in permanent dentition [2]. Establishing effective oral‐hygiene habits early in life is essential for long‐term oral health, as daily toothbrushing with fluoride toothpaste is fundamental for plaque control and caries prevention, making mechanical plaque removal a core component of preventive pediatric dentistry [3].

Toothbrushing is a learned motor behavior that relies on the integration of fine motor skills (manual dexterity, grip control, and brush manipulation) together with visual‐motor processes and procedural memory, developing over time through repeated practice [4]. Most children below 8–9 years lack the motor maturity and sustained attention required for thorough plaque removal, making parental supervision essential to ensure adequate oral hygiene [5]. Manual toothbrushes with conventional bristle designs further increase these demands, as they require precise angulation and controlled movements that exceed the motor and cognitive capacities of many young children [6]. Consequently, elevated plaque levels often persist in this age group despite regular brushing attempts.

To address this developmental barrier, electric toothbrushes and alternative manual toothbrush geometries have been developed to reduce the technical skill required for effective plaque removal. Clinical evidence from pediatric randomized‐controlled trials demonstrates that powered toothbrushes achieve significantly greater plaque reduction than manual toothbrushes, with individual studies reporting improvements ranging from near‐zero to over 35%, and pooled effects showing an average reduction of approximately 17% [7]. Additionally, specialized manual toothbrush designs such as triple‐headed brushes with simultaneous access to occlusal, buccal, and lingual surfaces have been proposed as a practical alternative for children with developing motor skills, as their configuration reduces the need for precise brush angulation and may facilitate more effective plaque removal compared with conventional single‐headed brushes [8]. However, comparisons of electric toothbrushes, triple‐headed manual brushes, and conventional manual brushes in the same pediatric population remain limited in the literature. Direct, within‐subject comparisons that simultaneously evaluate these three designs while accounting for individual developmental variation are therefore needed to guide individualized toothbrush selection in young children.

A critical gap exists in understanding how individual children's developmental characteristics predict their ability to use different toothbrush designs effectively. Although previous research has identified associations between fine motor skills and toothbrushing efficacy using the Five‐to‐Fifteen (5–15R) questionnaire in small samples [9], no studies have systematically assessed how a comprehensive profile of neurodevelopmental domains, including gross motor skills, fine motor skills, attention and executive functions, body perception, and other developmental characteristics, influences toothbrushing performance with different brush types. The 5–15R is a validated, parent‐reported instrument specifically designed to assess eight developmental domains in children aged 5–17 years and has demonstrated reliability in identifying developmental difficulties across both motor and cognitive‐behavioral dimensions [10]. Understanding whether children with specific developmental profiles (e.g., motor coordination difficulties and attention deficits) achieve differential plaque removal with electric versus triple‐headed versus conventional manual toothbrushes could inform clinically tailored recommendations and optimize oral‐hygiene outcomes in pediatric populations.

This study aimed: (1) to compare the efficacy of three different toothbrush types (conventional manual, triple‐headed manual, and rechargeable powered) in removing dental plaque in 7‐year‐old children using a randomized crossover design, and (2) to investigate whether parental‐reported developmental and behavioral characteristics (assessed via the 5–15R questionnaire) predict differential plaque‐removal efficacy with each toothbrush type.

## 2 Materials and Methods

### 2.1 Study Design and Setting

This study was designed as a single‐blind, randomized‐controlled, three‐period crossover clinical trial conducted between March and July 2024 at the Department of Paediatric Dentistry, Trakya University Faculty of Dentistry. Each participant used three toothbrush types sequentially: conventional manual toothbrush (Paro S39, Switzerland), triple‐headed manual toothbrush (Paro Superbrush, Switzerland), and rechargeable powered toothbrush (Oral‐B Junior 6+, Germany) (Figure 1), with 1‐week washout periods between each 2‐min brushing session (Figure 2). Developmental and behavioral characteristics were evaluated using the parent‐reported 5–15R [10] questionnaire. The study compared plaque‐removal efficacy among three toothbrush types and investigated the influence of children's developmental characteristics on toothbrushing performance.

**FIGURE 1:**
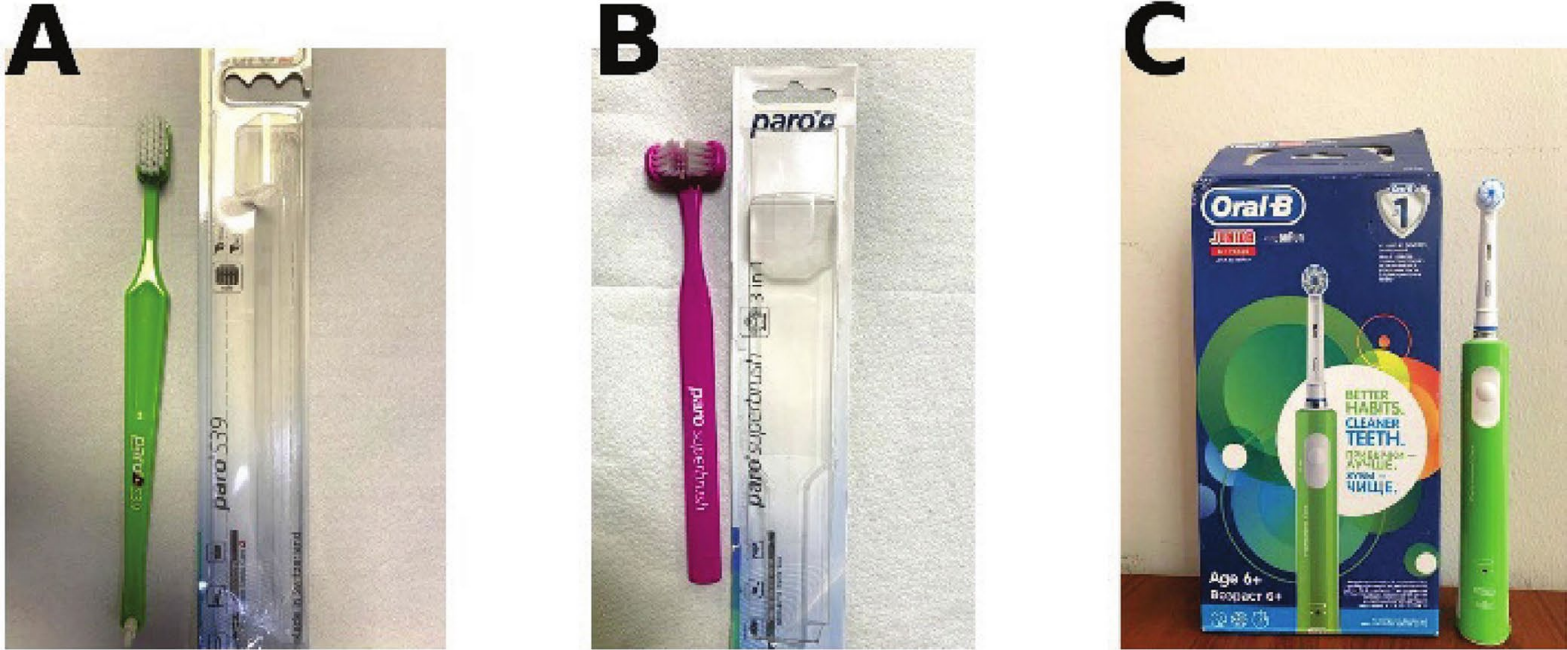
Toothbrush types evaluated for plaque‐removal efficacy in children. (A) Conventional single‐headed manual toothbrush; (B) triple‐headed manual toothbrush; and (C) powered toothbrush.

**FIGURE 2:**
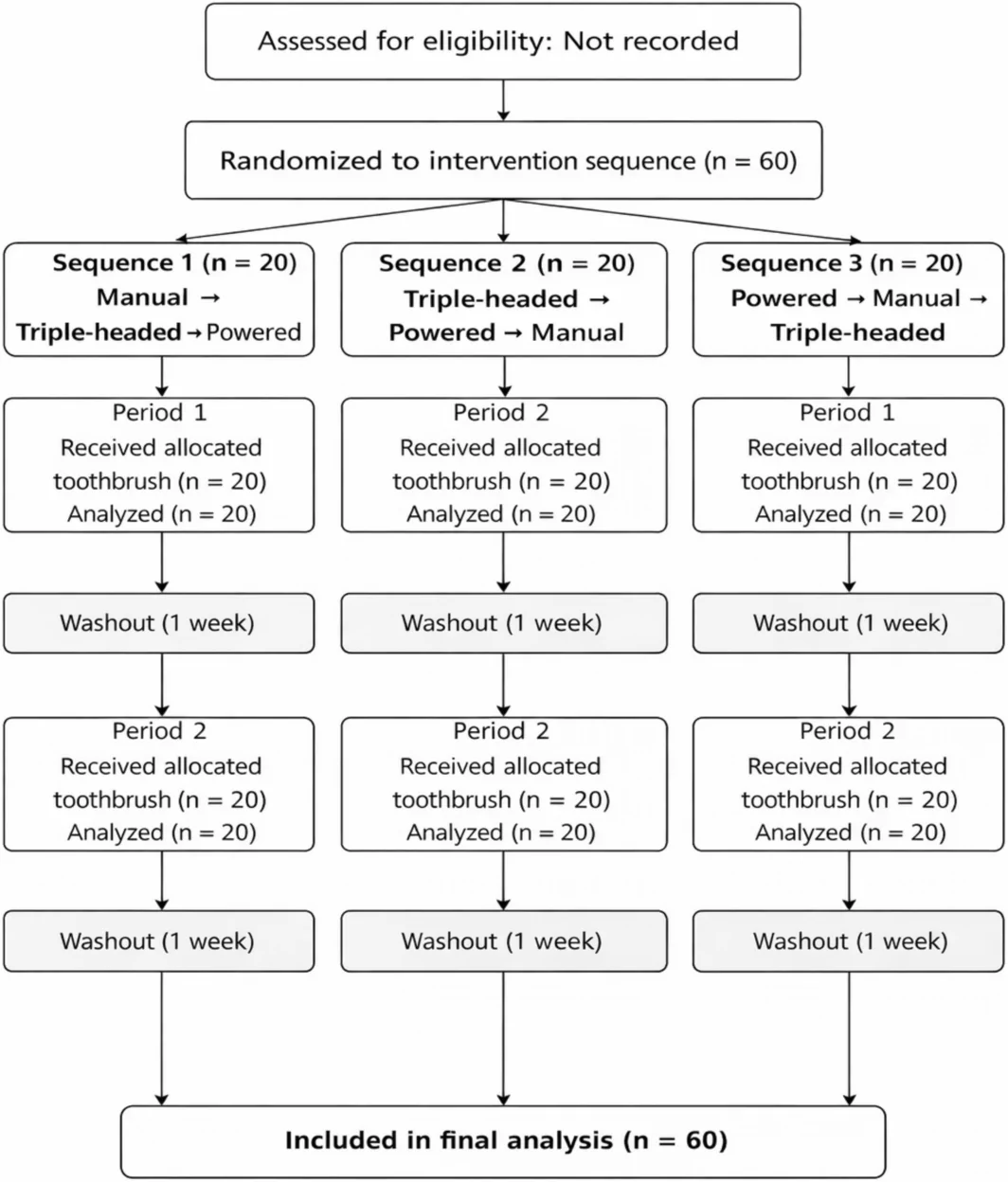
Flow diagram of the three‐period randomized crossover clinical trial. Sixty children were randomized to one of three intervention sequences. Each participant received all three toothbrush types across three study periods separated by washout intervals. All participants completed every period and were included in the final analysis (*n* = 60).

### 2.2 Sample Size and Participant Selection

Ethics approval for the study was obtained from the Trakya University Faculty of Medicine Non‐Interventional Scientific Research Ethics Committee (approval no. TÜTF‐GOBAEK 2019/393). The study was conducted in accordance with the principles of the Declaration of Helsinki. Written informed consent was obtained from the parents or legal guardians of all participating children, and verbal assent was obtained from each child prior to participation.

The sample size was determined a priori based on a pilot study by Durhan et al. [11], which used the modified Quigley–Hein Plaque Index as the primary outcome. Assuming a baseline plaque score of 2.71 ± 0.80 and a clinically meaningful difference in mean plaque reduction of approximately one‐third of a standard deviation between toothbrush types, with a two‐sided significance level of *α* = 0.05 and statistical power of 80%, a minimum of 60 participants was required. To account for the within‐subject correlation inherent to the three‐period crossover design and possible attrition, all 60 children completed each of the three toothbrushing conditions, yielding 180 paired observations. The study population consisted of 60 typically developing 7‐year‐old children (29 girls, 48.3%; 31 boys, 51.7%) recruited from patients (children) attending the university clinic. Inclusion criteria were: absence of any systemic or neurodevelopmental disorder that could affect motor function or oral‐hygiene performance (i.e., systemically healthy, ASA I), no carious lesions on permanent first molars, no major orthodontic malocclusion, fully erupted permanent first molars (Teeth 16, 26, 36, 46), completion of all three toothbrushing sessions, and parental completion of the developmental questionnaire. Children not meeting these criteria or whose parents declined participation were excluded.

### 2.3 Clinical Examination and Plaque Assessment Procedures

All examinations were performed in a dental treatment chair using a mirror and periodontal probe. Three toothbrush types were evaluated as described above. All participants used the same standardized fluoride toothpaste (Oral‐B Kids 3+, 500 ppm fluoride). Plaque was disclosed using GC Tri Plaque ID Gel (GC Corporation, Tokyo, Japan) as per the manufacturer's instructions. Baseline plaque levels were assessed using the modified Quigley–Hein Plaque Index [12] (0–5 scale: 0 = no plaque; 5 = plaque covering ≥ 2/3 of crown) and the Approximal Plaque Index [13] (presence/absence). Post‐brushing plaque was reassessed by a blinded examiner. Plaque scoring was restricted to the four fully erupted permanent first molars (Teeth 16, 26, 36, and 46). The modified Quigley–Hein Plaque Index was recorded on the buccal and lingual surfaces of these teeth, and the Approximal Plaque Index was assessed on their vestibular and oral approximal surfaces. Primary teeth and other permanent teeth were not included in the assessment, in order to standardize scoring across all participants.

### 2.4 Study Procedures and Randomization

After baseline plaque disclosure, participants were shown their plaque using a mirror. Brushing techniques were demonstrated verbally with a dental model: roll technique for manual brushes (bristles at gingival margin, swept toward occlusal surface), and slow passive motion across surfaces for the powered toothbrush. For the triple‐headed manual toothbrush, participants were instructed to position the brush correctly and use a back‐and‐forth motion, ensuring that the brush head simultaneously covered the occlusal, buccal, and lingual surfaces of the teeth. All brushing was supervised by an experienced pediatric dentist for 2 min. Post‐brushing plaque was assessed by a blinded examiner. Participants participated in three sessions with different toothbrush types. The first session toothbrush was determined by computer‐generated randomization with sealed allocation envelopes. One‐week washout periods were applied between sessions to minimize carryover effects. During each 1‐week washout period, participants continued their habitual home oral‐hygiene routine using their own conventional manual toothbrush, with no specific brushing technique imposed.

### 2.5 Examiner Calibration and Blinding

Examiner calibration was performed through discussion of pilot assessments on non‐study participants to establish scoring consensus. The outcome assessor remained blinded to toothbrush allocation, whereas the supervising dentist was aware due to the need to demonstrate toothbrush‐specific techniques.

### 2.6 Developmental and Behavioral Assessment

The 5–15R questionnaire is a standardized 181‐item instrument assessing eight developmental domains (gross motor skills, fine motor skills, perception and coordination, memory and learning, language and communication, attention and executive functions, social skills, and emotional/behavioral problems) [10]. Each item is rated on a 3‐point Likert scale (0 = does not apply, 1 = applies sometimes, 2 = applies).

At the time of this study, no validated Turkish version of the 5–15R was available. Therefore, the questionnaire was translated from English into Turkish and back into English using standard forward–backward translation procedures performed by a professional interpretation service, and the final Turkish version was reviewed and approved by the original developers of the instrument. The approved Turkish version was subsequently published on the official 5–15R website, where it was available as a free tool, and access to the online scoring system required prior registration.

Parents completed the questionnaire based on their observations of the participant's everyday functioning during the preceding 6 months, and responses were entered into the official electronic scoring system. Domain scores were calculated and interpreted using age‐specific normative percentiles as provided in the manual, with higher scores indicating greater developmental difficulties. For analytical purposes, participants were classified as having adequate or reduced performance in domains considered particularly relevant to independent toothbrushing ability, including gross motor skills, fine motor skills, attention and concentration, and perception of own body.

### 2.7 Statistical Analysis

Statistical analyses were performed using the IBM SPSS Statistics software package (version 23.0). The level of statistical significance was set at 0.05. The assumption of normal distribution for quantitative variables was assessed using the Shapiro–Wilk test. For comparisons between two independent groups, the independent samples *t*‐test was used for normally distributed quantitative variables, whereas the Mann–Whitney *U*‐test was applied for non‐normally distributed variables. For comparisons involving more than two normally distributed groups, one‐way analysis of variance (ANOVA) was used. Associations between categorical variables were examined using Pearson's chi‐squared test or Fisher's exact test, as appropriate. For comparisons involving more than two related non‐normally distributed groups, the Friedman test was used for quantitative variables, and Cochran's *Q*‐test was used for categorical variables. Because the primary analyses compared plaque reduction across the three toothbrush conditions using repeated‐measures testing, formal statistical tests for carryover and period effects (e.g., sequence‐by‐period interaction terms) were not performed; the 1‐week washout intervals were intended to minimize potential carryover between conditions.

### 2.8 Use of Artificial Intelligence Tools

Large language model–based tools (ChatGPT, OpenAI and Perplexity AI) were used to improve the clarity, grammar, and readability of the manuscript and for minor language editing. All outputs were critically reviewed and edited by the authors, who take full responsibility for the accuracy and integrity of the manuscript.

## 3 Results

### 3.1 Participant Characteristics

All 60 enrolled participants aged 7 years completed the study without dropouts. The sample comprised 29 girls (48.3%) and 31 boys (51.7%); 51 were right‐handed (85%) and nine left‐handed (15%). Forty‐six participants (76.6%) reported using a manual toothbrush at home, 14 (23.4%) used electric. Baseline plaque scores did not differ significantly by gender (girls: 2.995 ± 0.525; boys: 2.891 ± 0.548; *p* = 0.455), dominant hand (right: 2.926 ± 0.546; left: 3.027 ± 0.487; *p* = 0.605), or habitual toothbrush type (manual: 2.921 ± 0.503; electric: 3.008 ± 0.647; *p* = 0.596).

### 3.2 Plaque‐Removal Efficacy by Toothbrush Type

Mean plaque reduction differed significantly among the three brushes: conventional manual 1.310 ± 0.563, triple‐headed manual 1.802 ± 0.678, and powered 1.833 ± 0.705 (*p* < 0.001). Post hoc Tukey tests revealed both triple‐headed manual (mean difference = 0.491, *p* < 0.001) and powered brushes (mean difference = 0.522, *p* < 0.001) removed significantly more plaque than conventional manual brush. No significant difference was observed between triple‐headed manual and powered brushes (mean difference = 0.031, *p* = 0.963) (Table 1).

**TABLE 1:** Mean plaque reduction by toothbrush type (*n* = 60).

Toothbrush type	Mean plaque reduction ± SD
Conventional manual	1.310 ± 0.563
Triple‐headed manual	1.802 ± 0.678
Powered	1.833 ± 0.705

*Note:* Mean plaque reduction was compared among toothbrush types using one‐way analysis of variance (ANOVA) (*p* < 0.001). Post hoc pairwise comparisons were performed using Tukey's honestly significant difference test: triple‐headed manual versus conventional manual, mean difference = 0.491, *p* < 0.001; powered versus conventional manual, mean difference = 0.522, *p* < 0.001. No significant difference was observed between triple‐headed manual and powered toothbrushes (*p* = 0.963).

### 3.3 Tooth Surface–Specific Plaque‐Removal Efficacy

When analyzed by tooth surface, powered and triple‐headed brushes showed differential superior efficacy. Powered brush excelled on Tooth 16 buccal/lingual surfaces (buccal: 3.016 ± 1.631 vs. manual 2.266 ± 1.635, *p* = 0.001; lingual: 1.100 ± 1.068 vs. manual 0.550 ± 0.565, *p* < 0.001). Triple‐headed brush demonstrated superior performance on Tooth 36 buccal (2.033 ± 1.024 vs. manual 1.550 ± 1.032, *p* = 0.001) and Tooth 46 lingual (1.933 ± 1.363 vs. manual 0.983 ± 1.065, *p* < 0.001). Conventional manual brush showed consistently lowest efficacy across surfaces (Table 2).

**TABLE 2:** Mean plaque‐reduction scores (±SD) by toothbrush type for selected tooth surfaces.

Tooth	Surface	Conventional manual	Triple‐headed manual	Powered	*p* ^a^
16	Buccal	2.266 ± 1.635	2.550 ± 1.482	3.016 ± 1.631	0.001
16	Lingual	0.550 ± 0.565	0.850 ± 0.792	1.100 ± 1.068	< 0.001
36	Buccal	1.550 ± 1.032	2.033 ± 1.024	1.883 ± 1.102	0.001
46	Lingual	0.983 ± 1.065	1.933 ± 1.363	1.717 ± 1.289	< 0.001

Abbreviation: SD, standard deviation.

^a^ One‐way ANOVA.

### 3.4 Approximal Plaque Removal

Approximal plaque removal varied significantly by toothbrush type. Powered brush achieved highest efficacy: 78.3% complete removal on Tooth 16 vestibular surface (91.7% to 21.7%, *p* = 0.016), 73.3% on Tooth 36 vestibular (80% to 6.7%, *p* = 0.005), and 75% on Tooth 46 vestibular (85% to 10%, *p* = 0.001). Triple‐headed brush achieved moderate efficacy (50%–65% removal), whereas conventional manual achieved lowest efficacy (41.6%–50% removal). Cochran's *Q*‐test confirmed significant differences for vestibular surfaces of Teeth 16, 36, and 46, and oral surface of Tooth 36 (*p* ≤ 0.016) (Table 3).

**TABLE 3:** Approximal plaque presence before and after brushing according to toothbrush type (*n* = 60).

Tooth	Surface	Toothbrush type	Pre‐brushing (%)	Post‐brushing (%)	*p* ^a^
16	Oral	Conventional manual	60.0	21.7	0.452
		Triple‐headed manual	63.3	26.7	
		Powered	63.3	16.7	
	Vestibular	Conventional manual	98.3	50.0	**0.016**
		Triple‐headed manual	95.0	45.0	
		Powered	91.7	21.7	
26	Oral	Conventional manual	56.7	21.7	0.798
		Triple‐headed manual	58.3	18.3	
		Powered	55.0	18.3	
	Vestibular	Conventional manual	85.0	28.3	0.203
		Triple‐headed manual	83.3	23.3	
		Powered	90.0	18.3	
36	Oral	Conventional manual	86.7	58.3	**0.001**
		Triple‐headed manual	91.7	33.3	
		Powered	83.3	30.0	
	Vestibular	Conventional manual	83.3	36.7	**0.005**
		Triple‐headed manual	75.0	16.7	
		Powered	80.0	6.7	
46	Oral	Conventional manual	90.0	66.7	0.832
		Triple‐headed manual	93.3	38.3	
		Powered	91.7	33.3	
	Vestibular	Conventional manual	88.3	46.7	**0.001**
		Triple‐headed manual	86.7	21.7	
		Powered	85.0	10.0	

*Note: p* values refer to overall comparisons across the three toothbrush types. Bold *p*‐values indicate statistically significant differences (*p* < 0.05).

^a^ Cochran's *Q*‐test.

### 3.5 Influence of Participant Characteristics on Plaque‐Removal Efficacy

Subgroup analyses revealed minimal overall influence of participant characteristics. Girls showed marginally superior performance on specific surfaces with triple‐headed brush (Tooth 16 buccal: girls 2.620 ± 1.521 vs. boys 1.806 ± 1.621, *p* = 0.050; Tooth 46 buccal: girls 2.137 ± 1.156 vs. boys 1.580 ± 0.992, *p* = 0.049) and manual brush (Tooth 26 buccal: girls 2.551 ± 1.453 vs. boys 1.548 ± 1.479, *p* = 0.010), but performed equivalently with powered brush. Left‐handed participants outperformed right‐handed on Teeth 36 and 46 lingual surfaces with powered brush (Tooth 36: 2.111 ± 1.269 vs. 1.333 ± 1.032, *p* = 0.049; Tooth 46: 2.555 ± 1.509 vs. 1.372 ± 1.148, *p* = 0.009), but not with other brushes. Routine home toothbrush type did not significantly influence results (*p* ≥ 0.055 for all comparisons).

### 3.6 Developmental Characteristics and Toothbrushing Performance

For the manual toothbrush, mean plaque reduction did not differ between children with and without gross motor difficulties (1.261 ± 0.692 vs. 1.321 ± 0.537; *p* = 0.752). For the triple‐headed toothbrush, plaque reduction was lower in children with gross motor difficulties (1.465 ± 0.821) than in those without (1.877 ± 0.627), although the difference was not statistically significant (*p* = 0.069). In contrast, powered toothbrush use resulted in significantly lower plaque reduction in children with gross motor difficulties compared with those without (1.454 ± 0.673 vs. 1.918 ± 0.691; *p* = 0.048). No significant associations were observed between plaque‐reduction efficacy and other developmental domains across toothbrush types (all *p* ≥ 0.078; Table 4).

**TABLE 4:** Mean plaque‐reduction scores (±SD) according to gross motor development status by toothbrush type.

Toothbrush type	Gross motor difficulties	Mean plaque reduction ± SD	*p* ^a^
Conventional manual	Present	1.261 ± 0.692	0.752
	Absent	1.321 ± 0.537	
Triple‐headed manual	Present	1.465 ± 0.821	0.069
	Absent	1.877 ± 0.627	
Powered	Present	1.454 ± 0.673	**0.048**
	Absent	1.918 ± 0.691	

*Note:* No significant associations were observed for other developmental domains across toothbrush types (all *p* ≥ 0.078). Bold *p*‐values indicate statistically significant differences (*p* < 0.05).

^a^ Independent samples *t*‐test.

### 3.7 Safety and Compliance

All participants completed the study without adverse events. No soft tissue trauma, gingival abrasion, or adverse reactions were observed with any toothbrush type.

## 4 Discussion

This randomized crossover study compared plaque‐removal efficacy of three toothbrush types in 7‐year‐old children and investigated whether neurodevelopmental characteristics predict differential brushing performance. Both powered and triple‐headed manual toothbrushes removed significantly more plaque than conventional manual brushes, achieving approximately 40% greater plaque reduction. Notably, powered and triple‐headed brushes demonstrated equivalent overall efficacy, though with differential strengths on specific tooth surfaces. An exploratory observation was that children with gross motor‐skill difficulties showed lower plaque reduction with the powered toothbrush than children without such difficulties; this subgroup finding was based on a small number of participants and should be interpreted with caution. The powered toothbrush achieved the highest approximal plaque removal at 75% complete removal on posterior vestibular surfaces.

The superior plaque removal of powered toothbrushes compared with manual brushes is consistent with established pediatric literature [14] and with pediatric systematic reviews and meta‐analyses reporting a measurable plaque‐reduction advantage for powered over manual brushes in children [7, 15], including a 4 week trial by Davidovich et al. [16], which reported significantly greater plaque reduction with powered brushes. From a mechanistic perspective, conventional manual toothbrushes require precise 45 degree angulation and controlled sweeping motions, which are complex motor tasks that exceed the fine motor capabilities of most 7‐year‐old children. Powered toothbrushes automate these movements and allow effective plaque removal with simple guidance, thereby reducing developmental motor demands. Approximal cleaning is particularly challenging for children because of limited access and reduced manual dexterity. The observed 75% approximal plaque removal with powered toothbrushes exceeds typical manual brush performance and supports their use in children at high risk of caries. Improved interproximal plaque control with powered brushing has likewise been reported in younger populations [17].

To our knowledge, few studies have directly compared powered, triple‐headed, and conventional manual toothbrushes within the same pediatric sample using a crossover design; the equivalent efficacy of powered and triple‐headed brushes observed here therefore adds comparative evidence to a relatively under‐studied area. The plaque‐reduction efficacy of the triple‐headed brush observed here is in keeping with crossover evidence in young children, in whom the triple‐headed design removed significantly more plaque than a single‐headed brush across most surfaces [18], and with trials in children with limited motor or visual ability reporting comparable or superior plaque and gingival outcomes for triple‐headed brushes [19, 20]. The triple‐headed design is intended to reduce technical skill demands by allowing simultaneous contact with occlusal, buccal, and lingual surfaces. Our findings indicate that this design achieves overall plaque‐removal efficacy comparable to powered toothbrushes, although surface–specific differences were observed. The triple‐headed toothbrush showed superior performance on mandibular posterior surfaces, specifically Tooth 36 buccal and Tooth 46 lingual, whereas powered toothbrushes demonstrated greater efficacy on maxillary surfaces. From a clinical perspective, comparable efficacy supports the triple‐headed toothbrush as an important non‐electric alternative for families seeking cost‐effective options, wishing to avoid vibration and noise, or facing accessibility constraints.

A notable finding relates to gross motor‐skill difficulties and toothbrush efficacy. Among the 60 participants, 18.3% demonstrated gross motor difficulties according to the 5–15R questionnaire. Unexpectedly, within the powered‐toothbrush condition these children showed significantly lower plaque reduction than children without gross motor difficulties (1.454 ± 0.673 versus 1.918 ± 0.691, *p* = 0.048), despite the theoretical aim of powered toothbrushes to reduce motor demands. This should not be interpreted as manual brushes being superior for these children, since the between‐group difference reached statistical significance only in the powered condition and overall plaque reduction in this subgroup remained modest across all brush types. These observations are consistent with a growing body of work linking manual dexterity and motor coordination to toothbrushing efficacy in children: manual dexterity, encompassing both gross and fine motor skills, has been significantly associated with plaque‐removal efficacy in typically developing 7‐ to 12‐year‐olds [21], higher motor‐skill scores predict better oral hygiene in preschool children [22], and coordinative ability has been correlated with plaque levels in a large cohort of children and adolescents [23]. Importantly, a recent crossover trial in children with special needs found that toothbrush design influenced plaque removal independently of visual‐motor integration level [24], underscoring that the interaction between developmental ability and brush type is complex and warrants dedicated study. Several potential mechanisms may explain this finding. Children with motor difficulties may have difficulty stabilizing the powered toothbrush handle during oscillating and rotating movements, leading to suboptimal brush positioning. In addition, vibration related proprioceptive feedback may interfere with the tactile guidance required for effective positioning in children with motor difficulties. Furthermore, powered toothbrush use still requires sustained passive positioning and guidance, which depend on postural stability and fine motor control that may exceed the capabilities of children with motor coordination difficulties. The nonsignificant trend observed for triple‐headed toothbrushes (*p* = 0.069) suggests an intermediate pattern in which geometric design advantages may partially compensate for increased motor demands.

Clinically, these findings support developmentally informed toothbrush recommendations [25]. For most 7‐year‐old children, both powered and triple‐headed toothbrushes represent effective first‐line options. Screening for motor difficulties using the 5–15R questionnaire or similar tools may assist clinicians in selecting the most appropriate toothbrush for individual children. Those with motor difficulties may benefit from tailored instruction, enhanced parental involvement, or consideration of alternative toothbrush designs. Regardless of toothbrush type, effective plaque removal at this age remains highly dependent on supervision and guidance. This dependence is well documented: when children brush unsupervised to the best of their ability, substantial plaque remains, with 12‐year‐olds leaving plaque on approximately half of all tooth surfaces after brushing [26]. The brushing in our study was performed once under direct supervision, which standardizes technique but estimates the maximum achievable efficacy of each brush rather than its effectiveness during habitual, unsupervised home use, where the relative advantage of powered or triple‐headed brushes may differ.

Strengths of this study include the randomized crossover design controlling for inter‐individual variability, examiner blinding to reduce assessment bias, comprehensive developmental assessment using the 5–15R questionnaire, standardized supervised brushing procedures, and multiple outcome measures. Limitations include the single‐use assessment design, restriction to a single age group, lack of formal testing for period or carryover effects, and single center recruitment, which may limit generalizability. Small subgroup sizes further reduced power for analyses of developmental domains. The crossover design controls for inter‐individual variability, but period and carryover effects were not formally tested. Because plaque was disclosed and re‐scored at each single‐use session, true carryover from cumulative oral‐hygiene learning was not intended; nonetheless, a familiarization effect across periods cannot be excluded, as adaptation to a powered brush over repeated use has been reported to improve approximal plaque outcomes [24]. The randomized, counterbalanced sequence allocation is expected to distribute any such effect across treatment arms, but the absence of formal sequence and period testing is acknowledged as a limitation, and future trials should incorporate it.

In conclusion, powered and triple‐headed toothbrushes achieved significantly greater plaque reduction than conventional manual toothbrushes and demonstrated equivalent overall efficacy with complementary surface–specific strengths. The exploratory observation of reduced powered‐toothbrush efficacy in children with gross motor difficulties should be regarded as hypothesis‐generating and requires confirmation, but it raises the possibility that developmental characteristics merit consideration when recommending toothbrush type. These findings support individualized, developmentally informed approaches to oral‐hygiene guidance in pediatric dental practice.

## Author Contributions

E.B. designed the study and led manuscript drafting with E.B.M. and N.D. E.B.M. and N.D. contributed as clinical scientists and medical monitors. S.K. performed the statistical analysis. N.Ö. critically revised the manuscript for intellectual content. All authors approved the final version and agreed to be accountable for all aspects of the work.

## Funding

The authors have nothing to report.

## Ethics Statement

The study received ethical approval from the Trakya University Faculty of Medicine Non‐Interventional Scientific Research Ethics Committee (approval date: March 4, 2024; decision number: 04/31).

## Consent

Written informed consent was obtained from all parents after verbal and written explanation of the study purpose, procedures, benefits, and potential risks. An additional data protection and data‐sharing consent form was signed, informing participants that collected data could be reviewed by study quality monitors, ethics committees, and regulatory authorities as needed.

## Conflicts of Interest

The authors declare no conflicts of interest.

## Supporting information


**Data S1:** CONSORT 2025 checklist of information to include when reporting a randomized trial*.

## Data Availability

The data that support the findings of this study are available from the corresponding author upon reasonable request.

## References

[1] N. S. Jakubovics , S. D. Goodman , L. Mashburn‐Warren , G. P. Stafford , and F. Cieplik , “The Dental Plaque Biofilm Matrix,” Periodontology 2000 86, no. 1 (2021): 32–56, 10.1111/prd.12361. PMC9413593 33690911

[2] M. Kazeminia , A. Abdi , S. Shohaimi , et al., “Dental Caries in Primary and Permanent Teeth in Children's Worldwide, 1995 to 2019: A Systematic Review and Meta‐Analysis,” Head & Face Medicine 16, no. 1 (2020): 22, 10.1186/s13005-020-00237-z. PMC7541284 33023617

[3] S. Y. Han , C. L. Chang , Y. L. Wang , et al., “A Narrative Review on Advancing Pediatric Oral Health: Comprehensive Strategies for the Prevention and Management of Dental Challenges in Children,” Children 12, no. 3 (2025): 286, 10.3390/children12030286. PMC11941194 40150569

[4] R. Thavarajah , M. Kumar , A. A. Mohandoss , and L. T. Vernon , “Drilling Deeper Into Tooth Brushing Skills: Is Proactive Interference an Under‐Recognized Factor in Oral Hygiene Behavior Change?,” Current Oral Health Reports 2, no. 3 (2015): 123–128, 10.1007/s40496-015-0053-z. PMC4596060 26457238

[5] J. J. Winnier , S. S. Naik , R. Sharma , D. Patil , P. Gore , and S. Tandel , “Impact of Sustained Attention and Working Memory on Toothbrushing Efficacy in Typically Developing Children: A Cross‐Sectional Study,” Journal of the Indian Society of Pedodontics and Preventive Dentistry 43, no. 4 (2025): 523–529, 10.4103/jisppd.jisppd_302_25. 41235559

[6] E. Davidovich , R. A. Ccahuana‐Vasquez , H. Timm , J. Grender , and A. Zini , “Randomised Clinical Study of Plaque Removal Efficacy of an Electric Toothbrush in Primary and Mixed Dentition,” International Journal of Paediatric Dentistry 31, no. 5 (2021): 657–663, 10.1111/ipd.12753. PMC10015989 33225464

[7] A. Graves , T. Grahl , M. Keiserman , and K. Kingsley , “Systematic Review and Meta‐Analysis of the Relative Effect on Plaque Index Among Pediatric Patients Using Powered (Electric) Versus Manual Toothbrushes,” Dentistry Journal 11, no. 2 (2023): 46, 10.3390/dj11020046. PMC9955491 36826191

[8] S. M. Kalf‐Scholte , D. E. Slot , E. W. P. Bakker , and G. A. Van der Weijden , “Plaque Removal With Triple‐Headed vs Single‐Headed Manual Toothbrushes: A Systematic Review,” International Journal of Dental Hygiene 16, no. 1 (2018): 13–23. 10.1111/idh.12283 28544459

[9] A. C. Mafla , R. J. Benavides , P. Meyer , N. Giraudeau , and F. Schwendicke , “Association of Children's Toothbrushing and Fine Motor Skills: A Cross‐Sectional Study,” Brazilian Oral Research 36 (2022): e103, 10.1590/1807-3107bor-2022.vol36.0103. 35830146

[10] Gillberg Neuropsychiatry Centre, University of Gothenburg , “5–15‐R (FTF‐R) [Internet],” Gothenburg: University of Gothenburg (2026), https://www.gu.se/en/gnc/gncs‐resources/screening‐questionnaires‐and‐protocols/5‐15‐r‐ftf‐r.

[11] M. A. Durhan , M. Uluakay , and B. Kargul , “The Plaque Removal Effectiveness of Powered and Manual Toothbrush in Children: A Pilot Study,” Cumhuriyet Dental Journal 21, no. 4 (2018): 327–334.

[12] G. A. Quigley and J. W. Hein , “Comparative Cleansing Efficiency of Manual and Power Brushing,” Journal of the American Dental Association 65 (1962): 26–29. 10.14219/jada.archive.1962.0184 14489483

[13] T. G. Wilson, Jr. and K. S. Kornman , eds., Fundamentals of Periodontics, 2nd ed. (Quintessence Publishing Co Inc, 2003).

[14] F. Dağdeviren , G. A. F. Van der Weijden , C. P. L. Zijlstra , and D. E. Slot , “The Effectiveness of Power Versus Manual Toothbrushes on Plaque Removal and Gingival Health in Children—A Systematic Review and Meta‐Analysis,” International Journal of Dental Hygiene 23, no. 4 (2025): 682–702, 10.1111/idh.12915. PMC12516003 40739767

[15] E. Davidovich , S. Shafir , B. Shay , and A. Zini , “Plaque Removal by a Powered Toothbrush Versus a Manual Toothbrush in Children: A Systematic Review and Meta‐Analysis,” Pediatric Dentistry 42, no. 4 (2020): 280–287. 32847667

[16] E. Davidovich , R. A. Ccahuana‐Vasquez , J. Grender , H. Timm , H. Gonen , and A. Zini , “A 4‐Week Randomized Controlled Trial Evaluating Plaque and Gingivitis Effects of an Electric Toothbrush in a Paediatric Population,” International Journal of Paediatric Dentistry 34, no. 3 (2024): 246–255, 10.1111/ipd.13130. 37864381

[17] C. Erbe , H. Wehrbein , H. Timm , et al., “A Comparative Assessment of Plaque Removal and Toothbrushing Compliance Between a Manual and an Interactive Power Toothbrush Among Adolescents: A Single‐Center, Single‐Blind Randomized Controlled Trial,” BMC Oral Health 18, no. 1 (2018): 130, 10.1186/s12903-018-0588-1. PMC6091059 30075780

[18] K. Youcharoen , N. Thomngam , N. Aranya , Y. Wongphanthuset , M. Rungruangpattana , and N. Kaewsutha , “Plaque Removal Efficacy of Triple‐Headed Toothbrush in 4–6‐Year‐Old Children: A Randomized Crossover Study,” Journal of International Society of Preventive & Community Dentistry 11, no. 5 (2021): 546–552, 10.4103/jispcd.JISPCD_93_21. PMC8533035 34760799

[19] T. Tanaiadchawoot , A. Smutkeeree , S. Khamduangdao , and P. Kasemkhun , “Single vs Triple‐Headed Toothbrushes in Children With Special Needs: A Randomised Crossover Trial of Brushing Efficacy,” International Dental Journal 76, no. 2 (2026): 109388, 10.1016/j.identj.2025.109388. PMC12861218 41576728

[20] B. Roychowdhury , S. Jalihal , A. V. Ankola , et al., “Effectiveness of Triple‐Headed Versus Conventional Single‐Headed Toothbrushes on Gingival Health and Plaque Control Among Children and Adolescents With Visual Impairment: A Randomized Controlled Trial,” Special Care in Dentistry 45, no. 6 (2025): e70125, 10.1111/scd.70125. 41388883

[21] J. J. Winnier , S. S. Naik , A. Shetty , D. Patil , S. Tandel , and P. Gore , “Impact of Manual Dexterity on Toothbrushing Efficacy in Typically Developing Children: A Cross‐Sectional Study,” International Journal of Paediatric Dentistry 35, no. 3 (2025): 492–499, 10.1111/ipd.13260. 39192544

[22] L. Macote‐Orosco , A. Martín‐Vacas , M. M. Paz‐Cortés , M. R. Mourelle Martínez , and M. J. de Nova , “The Relationship Between Manual Dexterity and Toothbrushing Efficiency in Preschool Children: A Crossover Study,” Children 11, no. 12 (2024): 1498, 10.3390/children11121498. PMC11674593 39767927

[23] H. Kolbow , W. Kiess , C. Hirsch , M. Vogel , A. Schrock , and W. Elger , “The Influence of Coordinative Skills on the Oral Health of Children and Adolescents in Permanent Dentition,” Journal of Clinical Medicine 11, no. 21 (2022): 6472, 10.3390/jcm11216472. PMC9655693 36362700

[24] Z. Uçar and B. M. Akyıldız , “Plaque Removal Using Powered and Manual Toothbrushes in Children With and Without Attention Deficit Hyperactivity Disorder: A Randomized Controlled Trial,” BMC Oral Health 26, no. 1 (2026): 245, 10.1186/s12903-026-07881-y. PMC12980865 41691204

[25] D. R. Chua , S. Hu , Y. F. Sim , W. Lim , B. W. P. Lai , and C. H. L. Hong , “At What Age Do Children Have the Motor Development to Adequately Brush Their Teeth?,” International Journal of Paediatric Dentistry 32, no. 4 (2022): 598–606, 10.1111/ipd.12938. 34779540

[26] U. Weik , O. Cordes , J. Weber , et al., “Toothbrushing Performance and Oral Cleanliness After Brushing in 12‐Year‐Old Children,” JDR Clinical & Translational Research 7, no. 1 (2022): 71–79, 10.1177/2380084420975333. PMC8674791 33251929

